# Comparison of Oncocytic and Follicular Carcinoma of the Thyroid Without Initial Distant Metastasis

**DOI:** 10.1002/wjs.70018

**Published:** 2025-07-25

**Authors:** Pierre Puygrenier, Gabrielle Deniziaut, Sebastien Gaujoux, Jeanne Yen Khe Duong Vinh, Camille Buffet, Fabrice Menegaux, Nathalie Chereau

**Affiliations:** ^1^ Department of General, Visceral, and Endocrine Surgery Pitié‐Salpêtrière Hospital AP‐HP Paris France; ^2^ Sorbonne University Paris France; ^3^ Department of Pathological Anatomy and Cytology Pitié‐Salpêtrière Hospital Paris France; ^4^ Groupe de Recherche Clinique No 16 Thyroid Tumors Paris France; ^5^ Thyroid Disease and Endocrine Tumor Department Pitié‐Salpêtrière Hospital AP‐HP Paris France

**Keywords:** follicular thyroid carcinoma, Hurthle cell carcinoma of the thyroid, oncocytic thyroid carcinoma, thyroid carcinoma

## Abstract

**Objectives:**

Oncocytic thyroid carcinoma (OTC) is recognized as a distinct entity of follicular thyroid carcinoma (FTC), and its prognosis has traditionally been considered poor. However, the available data on this topic remain limited and highly heterogeneous. The objective of this study was to investigate the clinicopathological features and prognosis of patients with OTC and compare them with those of patients with FTC.

**Methods:**

A retrospective comparative study of all patients who underwent total thyroidectomy for OTC or FTC in a high‐volume endocrine surgery center between January 2005 and December 2021. Demographic and clinicopathological characteristics, complications, and recurrence rates were compared using univariate and multivariate regression analyses.

**Results:**

In total, 347 patients, 72% female, median age 48 years (35–61 years) were enrolled, including 123 with OTC and 224 with FTC. Patients were significantly older in the OTC group than in the FTC group (54.7 vs. 44.5 years, respectively, *p* = 0.001). With respect to other demographic, clinical, and pathological characteristics, no statistically significant differences were observed between the two groups. The median follow‐up duration was 5.6 years (3.2–10.5 years). Eighteen patients (5.2%) experienced recurrence, including 7 (5.7%) in the OTC group and 11 (4.9%) in the FTC group. No difference in recurrence‐free survival was observed between the OTC and FTC groups (*p* = 0.47). In multivariate analysis, histological type was not associated with the risk of recurrence.

**Conclusion:**

Patients withOTC have the same recurrence rate as do patients with FTC . It does not appear justified to propose a different therapeutic management for this histological type, and a stepdown management could be proposed.

## Graphical abstract

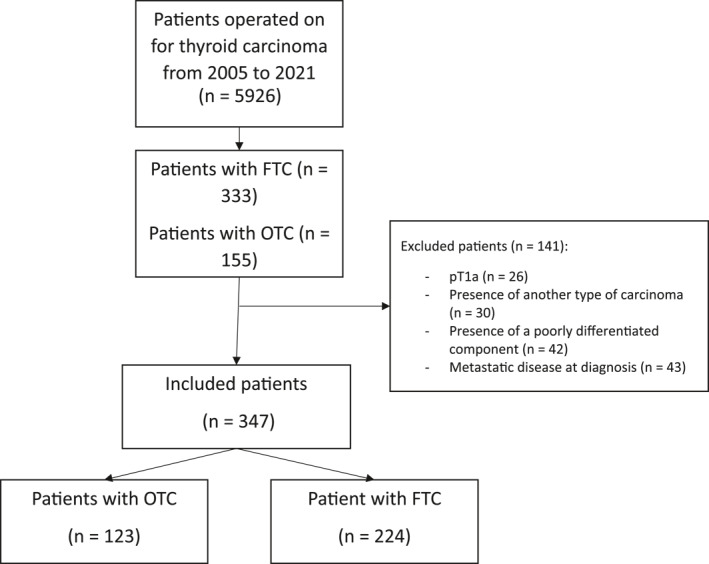

## 1 Introduction

Follicular thyroid carcinoma (FTC) and oncocytic thyroid carcinoma (OTC) are rare subtypes of well‐differentiated thyroid cancer, representing approximately 4% and 2% of all well‐differentiated thyroid cancers, respectively [1]. Although OTCs were previously considered as a variant of FTCs according to the 2004 World Health Organization (WHO) classification of endocrine tumors, the 2017 WHO classification ultimately distinguished between FTC and “Hürthle cell carcinomas” [2]. This distinction was further confirmed in the 2022 WHO classification, which replaced the term “Hürthle cells” with “oncocytic cells” to avoid any histopathological confusion [3]. The origin of this separation is partly linked to the genetic characteristics of OTCs, characterized by chromosomal instability with the loss of one arm of most chromosomes and the duplication of chromosomes 5 and 7. These anomalies are not observed in FTCs [4, 5, 6]. Molecular analysis has also revealed mitochondrial DNA mutations, particularly in genes associated with complex I of the respiratory chain [7]. Furthermore, RAS1 gene mutations, which are the most frequently observed mutations in FTCs (approximately 50%), are found in only 10%–15% of OTCs [4, 5].

OTCs have been classically considered to have a poorer prognosis than FTCs because of a higher prevalence of lymph node involvement and distant metastases, a more frequent multifocal character, and decreased sensitivity to iodine‐131 [8, 9, 10, 11]. However, the results regarding survival comparisons between patients with FTC and those with OTC remain controversial in the literature [12, 13, 14, 15, 16, 17]. Similarly, recurrence‐free survival is highly heterogeneous across studies, with recurrence rates ranging from 14% to 44% for patients with OTC and from 3% to 43.5% for patients with FTC [10]. These findings may be explained by studies bias with small sample sizes due to the relative rarity of these cancers [12, 18, 19, 20, 21] and temporal dispersion reflecting the heterogeneity of management practices over time [12, 18, 19, 20, 21].

The objectives of this study were to compare the clinicopathological features and outcomes between patients with FTC and OTC, including a comparison of recurrence‐free survival (RFS) and cancer‐specific death.

## 2 Patients and Methods

### 2.1 Patients

This is a single‐center retrospective comparative study in which data were prospectively collected at a high‐volume endocrine surgery center. Data were retrieved from a prospectively maintained institutional database approved by the Institutional Research Ethics Committee (Ethics Committee of Sorbonne University in Pitié‐Salpêtrière Hospital, registration number 20200115171338). According to French law, retrospective studies built exclusively on data do not need the approval of “committees for the protection of individuals”. All included patients were individually informed beforehand about the use of their personal data. This study was performed in accordance with the ethical principles of the Declaration of Helsinki.

All consecutive adult patients (≥ 18 years old) who underwent total thyroidectomy for FTC or OTC between January 1, 2005, and December 31, 2021, were included. When needed, patients charts were reviewed. An expert pathologist reviewed all the histological reports to confirm either FTC or OTC diagnosis.

Patients with another type of associated carcinoma, poorly differentiated component and distant metastatic stage cancer M1 were excluded. Patients with microfollicular or micro‐oncocytic carcinoma classified as pT1a in the pathological report were excluded because of their highly favorable prognosis. Patients lost to follow‐up immediately after their initial treatment (surgery or radioactive iodine therapy when indicated) were also excluded.

### 2.2 Surgery and Radioactive Iodine Therapy

The operative management was homogeneous. Thyroidectomy (TT or lobectomy) involves therapeutic lymph node (LN) dissections in any patient with suspected LN metastasis preoperatively. In the absence of suspicious lymph nodes, prophylactic central compartment dissection was occasionally performed based on institutional practice. Decisions regarding completion thyroidectomy following an initial lobectomy were made during specialized multidisciplinary team meetings on the basis of the current recommendations [22]. All patients with FTC or OTC > 10 mm had an indication for TT.

We collected patient demographic data (age, sex, and body mass index [BMI]) and operative details (TT in one‐ or two‐stage and LN dissection). The age of patients was divided into two groups (< 55 years and ≥ 55 years), as it constitutes an independent prognostic factor in thyroid cancer, and the threshold of 55 years was adopted by the 2017 TNM classification to distinguish these groups [23, 24].

The following data were obtained from pathological examination: tumor size, number of tumors, bilaterality, multifocality, extrathyroidal extension, and vascular invasion (limited vascular invasion < 4 foci vs. extensive vascular invasion ≥ 4 foci), pathological tumor staging in accordance with the eight edition of the American Joint Committee on Cancer Pathological Tumor Node Metastasis (TNM) staging system [24], and lymph node dissection.

Indications and activity of postoperative radioactive iodine therapy were determined during the multidisciplinary meetings in accordance with institutional guidelines: patients with high‐risk carcinoma (according to the ATA classification) received 100 mCi (3.7 GBq) of radioactive iodine after thyroid hormone withdrawal for 4 weeks. Lower‐risk (intermediate) patients received 30 mCi (1.1 GBq) or 100 mCi recombinant human TSH depending risk of recurrences, and low‐risk carcinomas were managed without radioiodine treatment.

### 2.3 Follow‐Up and Recurrence

Follow‐up was standardized at 6 and 12 months and then annually. The tests included physical examination, cervical ultrasound, basal thyroglobulin (TG) measurement or TG stimulated by recombinant TSH, and antithyroglobulin antibody (anti‐TG Ab) testing. For high‐risk patients, a cervical‒thoracic CT scan was performed when necessary. After 6 years of follow‐up in patients free of disease, periodic correspondence with the patients or their referring physicians is conducted every 3 years.

Recurrence was defined as follows: (1) an isolated and repeated elevation of serum thyroglobulin (Tg) levels—typically > 10 ng/mL after stimulation (using Thyrogen or LT4 withdrawal) or elevated ultrasensitive Tg under suppression therapy—confirmed on at least two consecutive measurements; (2) the appearance of proven locoregional recurrence (LRR) in the thyroid bed, soft tissues, or cervical lymph nodes; or (3) the development of distant metastases. The method used for Tg assessment—whether stimulated (using Thyrogen or LT4 withdrawal), suppressed, or based on ultrasensitive assays—was determined by the clinical context and institutional practices at the time. Imaging (RAI scan, cervical ultrasound, or PET‐CT) was used when indicated to confirm structural recurrence.

## 3 Comparison Between FTC and OTC

Data from patients with FTC were compared with those from patents with OTC treated during the same study period (2005–2021). We compared clinicopathological characteristics and outcomes in terms of disease recurrence, the development of distant metastases, recurrence‐free survival (RFS), and cancer‐specific death.

### 3.1 Statistical Analysis

Quantitative values are presented as the medians with interquartile ranges (IQRs), and qualitative values are presented as the counts and percentages. Quantitative variables were compared using the Mann–Whitney test, whereas qualitative variables were compared via the chi‐squared test and Fisher's exact test.

RFS was estimated using the Kaplan–Meier method with a 95% confidence interval. The log‐rank test was used to compare survival curves obtained through the Kaplan–Meier method. RFS was defined as the time interval between the date of surgery and the date of recurrence diagnosis. Univariate Cox proportional hazards models were used to identify independent factors influencing recurrence. A multivariable Cox proportional hazards model was used to examine factors associated with the likelihood of recurrence in the entire cohort. A two‐sided significance level of 0.05 was used for all the statistical tests. Analyses were performed using SAS v. 9.4 (SAS Institute, Cary, NC, USA).

## 4 Results

**FIGURE 1:**
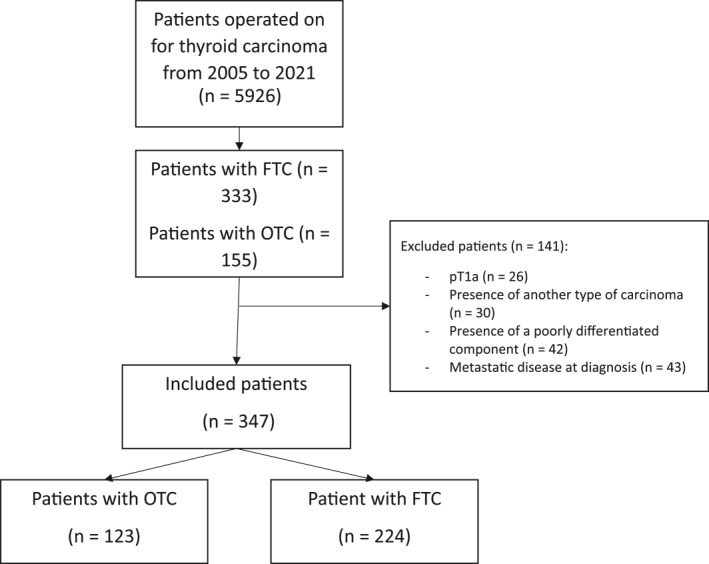
Flowchart.

Between January 1, 2005, and December 31, 2021, 347 patients (72% female, median age 48 years [range: 35–61 years]) were enrolled, with 123 with OTC and 224 with FTC (Figure 1).

Patients were significantly older in the OTC group than in the FTC group, with median ages of 54.7 years (IQR, 44.0–63.8) and 44.5 years (IQR, 32.3–58.7), respectively (*p* = 0.001). The proportion of patients aged over 55 years was also significantly greater in the OTC group (50% vs. 32% and *p* = 0.001). No other statistically significant demographic differences were observed between the two groups (Table 1).

**TABLE 1:** Demographic characteristics, surgery, and radioactive iodine therapy of patients with OTC or FTC.

	OTC	FTC	Total	*p* value
	*n* = 123	*n* = 224	*n* = 347	
Age, years (IQR)	54.7 (44.0–63.8)	44.5 (32.3–58.7)	47.9 (35.4–60.6)	**0.001**
Age group, years				
≥ 55 years	61 (49.6)	71 (31.7)	132 (38)	**0.001**
BMI, kg/m² (IQR)	24.8 (22.7–28.7)	24.6 (21.6–28.4)	24.7 (22.0–28.6)	0.148
Total thyroidectomy				0.06
In one‐step	39 (32)	95 (42)	134 (39)	
In two‐step	84 (68)	129 (57.6)	213 (61)	
Lymph node dissection	39 (32)	91 (41)	130 (38)	0.14
Isotopic ablation	123 (100)	224 (100)	347 (100)	

*Note:* Qualitative variables are expressed as proportions. Values in parentheses are percentages. Quantitative variables are expressed as medians (interquartile range).

Abbreviations: BMI, body mass index; FTC, follicular thyroid carcinoma; IQR, interquartile range; OTC, oncocytic thyroid carcinoma.

All patients underwent total thyroidectomy (TT) in one stage (38%) or two stages (62%). Cervical lymph node (LN) dissection was performed in 130 patients (37.5%), with no significant difference between the two groups (*p* = 0.14). Among these, 97.7% were prophylactic central compartment dissections and 2.3% were therapeutic central and lateral dissections. When prophylactic dissection was performed, lymph node involvement was found in only 1.6% of cases. By tumor subtype, prophylactic dissection was performed in 31.7% of patients with oncocytic thyroid carcinoma (OTC) and 39.3% of those with conventional follicular thyroid carcinoma (FTC). Among patients who underwent prophylactic dissection, lymph node metastasis was found in only 1 patient with OTC and 1 patient with FTC. When considering all lymph node dissections (both prophylactic and therapeutic), lymph node metastasis was identified in 5.1% of OTC cases and 3.3% of FTC cases, with no significant difference between the two histological subtypes (*p* = 0.64). A greater proportion of patients with OTC received the 100 mCi dose compared to patients with FTC (73% vs. 65%), but the difference was not statistically significant (*p* = 0.1) (Table 1).

There was no significant difference in the median tumor size (*p* = 0.8) or the pTNM stage between the two groups. However, a significant difference was observed in the risk of recurrence according to the 2015 ATA guidelines [22] (*p* = 0.045), with a greater proportion of intermediate‐risk patients in the OTC group (59.3% vs. 46%) than in the FTC group (Table 2). There was no significant difference in LN involvement or capsular invasion between the two groups (Table 3).

**TABLE 2:** Histopathological characteristics of patients with OTC or FTC.

	OTC	FTC	Total	*p* value
	*n* = 123 (35.4)	*n* = 224 (64.6)	*n* = 347	
Tumor size, mm (IQR)	32 (24–42)	32 (23–43)	32 (24–43)	0.80
Tumor size (%)				0.55
> 40 mm	32 (26)	65 (29)	97 (28)	
pT stage, TNM				0.71
T1b	23 (18.7)	47 (21)	70 (20.1)	
T2	68 (55.3)	112 (50)	180 (51.9)	
T3	31 (25.2)	64 (28.6)	95 (27.4)	
T4	1 (0.8)	1 (0.4)	2 (0.6)	
pN stage, TNM				0.62
N0	41 (33.4)	86 (38.4)	127 (36.6)	
N1	2 (1.6)	3 (1.3)	5 (1.4)	
Nx	80 (65)	135 (60.3)	215 (62)	
Recurrence risk^a^				**0.045**
Low risk	49 (39.8)	117 (52.2)	166 (47.8)	
Intermediate risk	73 (59.3)	103 (46)	176 (50.7)	
High risk	1 (0.8)	4 (1.8)	5 (1.4)	
Multifocality	16 (13)	35 (15.6)	51 (14.7)	0.51
Bilaterality	12 (9.8)	35 (15.6)	47 (13.5)	0.13
Capsular invasion				0.22
Miminally invasive	77 (81.1)	120 (87)	197 (84.5)	
Widely invasive	18 (18.9)	18 (13)	36 (15.5)	
Missing data	28	86	114	
Vascular invasion	74 (65)	80 (50.6)	154 (56.6)	0.22
Missing data	9	66	75	
Type of vascular invasion				0.71
Foci < 4	50 (75.8)	58 (78.4)	108 (77.1)	
Foci ≥ 4	16 (24.2)	16 (21.6)	32 (22.9)	
Missing data	8	6	14	
Extrathyroidal extension	3 (2.4)	11 (4.9)	14 (4)	0.39

*Note:* Qualitative variables are expressed as proportions. Quantitative variables are expressed as medians (interquartile range).

Abbreviations: cm, centimeter; FTC, follicular thyroid carcinoma; IQR, interquartile range; mm, millimeter; OTC, oncocytic thyroid carcinoma; pN, regional lymph node; pT, primary tumor.

^a^ Recurrence risk is calculated based on the 2015 American Thyroid Association classification.

**TABLE 3:** Univariate and multivariate analyses of factors associated with disease recurrence.

	Univariate analysis		Multivariate analysis	
Variable	HR (95% CI)	*p* value	HR (95% CI)	
Age ≥ 55 years	4.31 (1.52–12.26)	**0.0006**	1.27 (0.20–8.01)	0.8
Male sex	1.21 (0.43–3.43)	0.72		
Tumor size > 40 mm	1.81 (0.69–4.76)	0.23		
Multifocality	0.76 (0.17–3.32)	0.71		
Bilaterality	1.39 (0.40–4.85)	0.60		
Risk stratification^a^				
Intermediate	0.77 (0.28–2.07)	0.60		
High	5.92 (0.74–47.37)	0.09		
OTC	0.69 (0.26–1.83)	0.46		
Vascular invasion	2.49 (0.53–11.83)	0.25		
Vascular invasion with ≥ 4 foci	6.43 (1.17–35.2)	**0.03**	0.70 (0.05–9.19)	0.8
Wide capsular invasion	10.11 (1.98–52.78)	**0.006**	10.7 (1.01–113)	**0.049**
Extrathyroidal extension	6.04 (1.72–21.10)	**0.05**	3.4 (0–6.2)	0.08

*Note:* HR, hazard ratio; 95% confidence interval.

Abbreviation: OTC, oncocytic thyroid carcinoma.

^a^ Risk stratification based on the 2015 American Thyroid Association classification.

There was also no significant difference in vascular invasion between the OTC and FTC groups (65% versus 51%, respectively [*p* = 0.22]). Specifically, fewer than 4 emboli were observed in 77% of patients, with no significant difference between the two groups. No other significant difference was observed between the two groups (Table 2).

**FIGURE 2:**
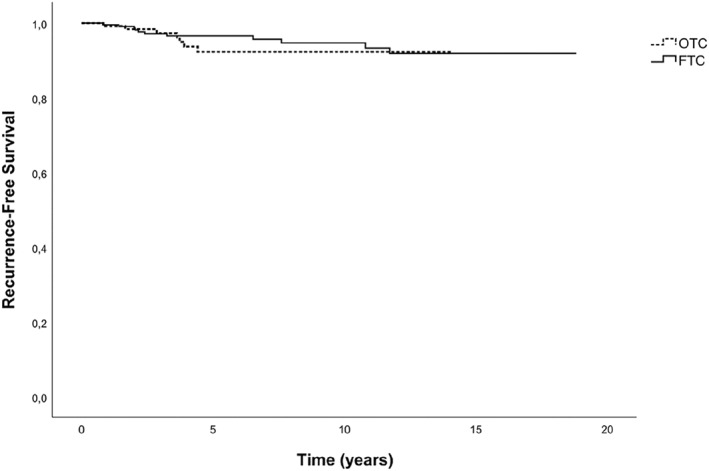
Comparison of RFS between patients with OTC and FTC.

Over a median follow‐up duration of 5.61 years (IQR, 3.23–10.53), 18 patients (5.2%) experienced recurrence. Among these patients, 8 presented with an isolated and repeated increase in serum TG levels requiring the administration of a second therapeutic dose of iodine‐131, 8 patients experienced LRR, and 2 patients developed pulmonary metastases. Specifically, among patients with OTC, 7 (5.7%) experienced recurrence (3 cases of isolated TG elevation and 4 cases of LRR), whereas 11 patients (4.9%) with FTC (5 cases of isolated TG elevation, 4 cases of LRR, and 2 cases of metastatic disease) experienced recurrence. The median RFS was 3 years (95% CI, 2.5–3.5) for OTC and 3.9 years (95% CI, 3.6–4.4) for FTC, with no significant difference (*p* = 0.22). The 5‐year and 10‐year RFS rates for patients with OTC were 92.3%, whereas they were 96.5% and 94.6%, respectively, for patients with FTC. Kaplan–Meier analysis of RFS (Figure 2) revealed no significant difference between the two groups (*p* = 0.467).

Six patients (1.7%) died from thyroid carcinoma, including 3 with OTC and 3 with FTC. Cancer‐related deaths were 98.7% for FTC and 97.6% for OTC.

In the univariate analysis, age ≥ 55 years, vascular invasion with ≥ 4 foci, widely invasive tumor characteristics, and extrathyroidal extension were associated with an increased risk of recurrence. In the multivariate analysis, after adjusting for other variables in the Cox PH model, the histological type of cancer (OTC or FTC) was not associated with recurrence, and the only significant factor associated with recurrence was the wide capsular invasion (HR 10.7; 95% CI, 2.5–3.5; and *p* = 0.05).

## 5 Discussion

Our results did not reveal any differences in RFS between the two groups (*p* = 0.47), with 7 patients (5.7%) experiencing recurrence in the OTC group compared with 11 patients (4.9%) in the FTC group.

Although the characteristics of the two groups were comparable, the median age was significantly greater in the OTC group than in the FTC group (54.7 years vs. 44.5 years, respectively, *p* = 0.001). This difference has also been reported in the literature [8, 10, 12, 14, 15, 17, 21, 25]. Additionally, a significant difference was observed in risk stratification according to the 2015 ATA guidelines [22] (*p* = 0.05), with a higher proportion of intermediate‐risk recurrence (59% vs. 46%) in the OTC group. Although these two criteria have been identified as associated with poor prognosis [22, 23], they did not lead to differences in RFS in this study, reinforcing our observation that OTCs do not present a worse RFS than do FTCs. Few studies support these findings [14, 15], notably with an overall recurrence rate of 3%, reported in the study by Kim et al. [14]. This contradicts the results reported by a previous study who reported a recurrence rate of 17% for OTCs compared with 8% for FTCs (*p* = 0.037) [16]. More recently, a systematic review of the literature revealed highly heterogeneous results, with recurrence rates ranging from 14% to 44% for OTCs and from 3% to 43.5% for FTCs [10]. Furthermore, the four studies based on the SEER database, which includes the largest number of patients with OTCs, did not provide recurrence data [8, 13, 25, 26].

This study also sought to identify risk factors associated with recurrence. According to the multivariate analysis, the only significant factor associated with recurrence was the widely invasive nature of the tumor (HR 10.7; 95% CI, 2.5–3.5; and *p* = 0.049). Conversely, there was no association between the histological type of cancer and recurrence. This finding contrasts with the study by Wenter et al., where the OTC histological type was identified as the sole factor associated with recurrence [16]. For Kim et al., the risk factors for recurrence were tumor size and lymph node involvement [14].

These contradictions regarding the prognosis of OTCs and FTCs could be attributed to the study period. The inclusion of patients from earlier periods, as in Wenter et al.’s study (1997–2017 vs. 2005–2021 in our study) [16], could have influenced recurrence rates. This impact of the study period on prognosis was reported by Nagar et al., who noted a reduction in OTC mortality over the past 35 years, with an increase in 5‐year survival of 21.7% and 10‐year survival of 21.3% between 1975 and 2009. Thus, although the survival data initially differed between the two groups, they ultimately became comparable over time. However, the precise reasons for this improvement in survival remain unclear [13].

From a histological perspective, an interesting finding of this study is the lack of difference in the rate of vascular invasion between the OTC and FTC groups (*p* = 0.22). The number of emboli was also considered in our study, as vascular invasion is well established to significantly influence survival for both OTCs and FTCs [27, 28, 29, 30, 31]. No significant difference in the extent of vascular invasion was observed between the two groups (*p* = 0.71). To our knowledge, only one other study has specifically compared the extent of vascular invasion between OTCs and FTCs. That study demonstrated a trend toward more extensive vascular invasion in patients with OTCs (33% vs. 19% and *p* = 0.07) and a significant prognostic impact of vascular invasion, with reduced RFS in patients with extensive vascular invasion [21].

With respect to other demographic, clinical, and pathological characteristics, no statistically significant differences were observed between the two groups. Specifically, data considered to have a significant prognostic impact (tumor size, extent of capsular invasion, and extrathyroidal extension) did not differ between OTCs and FTCs.

This study also evaluated lymph node involvement in OTCs and FTC. No significant difference in lymph node involvement was detected between OTCs and FTCs (5.1% vs. 3.3%, *p* = 0.64). OTCs are often considered to have higher rates of lymph node involvement than do FTCs [9, 10]. However, the literature on this topic is highly heterogeneous, with positive dissection rates ranging from 2.6% to 22% for OTCs [15, 16, 21, 26, 32, 33, 34]. Our study provides new evidence to challenge this notion, with OTC lymph node involvement rates falling at the lower end of the range. In particular, prophylactic central compartment dissection was performed in 31.7% of patients with OTC and 39.3% of those with FTC, despite current guidelines not recommending routine prophylactic dissection for either histologic type due to their low lymphotropic nature [35]. Among these patients, lymph node metastases were identified in only 1 patient in each group, further reinforcing the idea that both tumor types have a limited tendency for lymphatic spread.

All patients included in this study underwent total thyroidectomy in one or two stages according to the practices in our hospital during the inclusion period (2005–2021). However, this approach is evolving toward therapeutic de‐escalation, as reflected in current recommendations [22, 35], tailored to the specific nature and prognostic evaluation of the tumor [22, 35, 36]. For patients at low risk of recurrence, thyroid lobectomy appears sufficient for managing both OTCs and FTCs [37]. Since 2023, we have modified its practices in accordance with the French recommendations issued by SFE/AFCE/SFMN in 2022 [35].

This study has several limitations. First, this was a retrospective study conducted over a long inclusion period, and some pathological data were missing for certain patients. Second, our study is limited by the relatively small sample size, which may reduce the statistical power to detect subtle differences between the two carcinoma types. Third, treatment strategies evolved over the study period, reflecting a shift from a standardized, aggressive approach toward a more personalized, risk‐adapted management strategy aimed at therapeutic de‐escalation. However, this evolution occurred uniformly across both groups (OTC and FTC), with patients treated similarly within each time frame. Therefore, this institutional evolution in practice is unlikely to have introduced a treatment‐related bias between the two groups. Finally, due to the rarity of oncocytic thyroid carcinomas and the low number of recurrences, it is unlikely that a prospective study with a prolonged inclusion period could be realistically conducted to further investigate the optimal management of OTCs.

One strength of this study is that the various histopathological characteristics were carefully reviewed by the author and a pathologist specializing in thyroid pathology to minimize classification errors and avoid potential bias from histopathological changes during the inclusion period.

## 6 Conclusion

Based on this study, there does not appear to be a difference in prognosis between patients with OTC and those with FTC. Although they differ histologically and molecularly, we consider that therapeutic indications should not differ between these two types of thyroid cancer. Despite the use of an extensive surgical approach and radioactive iodine therapy, the recurrence rate remains high, similar to that of high‐risk papillary thyroid carcinoma, which requires careful ongoing surveillance.

## Author Contributions

**Pierre Puygrenier:** conceptualization, methodology, data curation, formal analysis, investigation, software, validation, visualization, writing – original draft. **Gabrielle Deniziaut:** conceptualization, data curation, project administration, visualization. **Sebastien Gaujoux:** validation, visualization, writing – review and editing. **Jeanne Yen Khe Duong Vinh:** conceptualization, data curation, project administration, visualization. **Camille Buffet:** project administration, visualization, supervision, writing – review and editing. **Fabrice Menegaux:** conceptualization, project administration, writing – review and editing, supervision, visualization. **Nathalie Chereau:** conceptualization, data curation, formal analysis, investigation, methodology, software, visualization, validation, supervision, writing – review and editing, writing – original draft.

## Ethics Statement

This study was approved by the Institutional Research Ethics Committee (Ethics Committee of Paris region VI in Pitié‐Salpêtrière Hospital, registration number 20200115171338). The authors followed the STROBE statement and used the STROBE checklist for observational studies (submitted with the manuscript).

## Conflicts of Interest

The authors declare no conflicts of interest.

## Data Availability

Research data are not shared.
